# 

**Published:** 1890-07

**Authors:** 


### CONTENTS.

### COMMUNICATIONS.

Thirty-Fifth Annual Meeting of the Michigan State Dental Association ........ 317
A Consideration of Dental Patents ........ 338
A Case in hand ........ 345
Reduction of Fracture of Superior Maxillary ........ 357
Does Our Profession Confer all the Benefits it Should? ........ 358
Invitation to the Tenth Medical Congress ........ 363

### EDITORIAL.

A Dental Gathering—1893 ........ 365
Notices ........ 367
Bibliographical ........ 367
Obituary ........ 368

